# Seroprevalence of pestivirus in Eurasian tundra reindeer in Finland, Sweden, Norway, Iceland and Russian Federation

**DOI:** 10.1080/20008686.2019.1682223

**Published:** 2019-10-29

**Authors:** Anna Omazic, Caroline Aurosell, Valery Fedorov, Åsa Hagström, Juha Kantanen, Mikael Leijon, Torill Mørk, Christine S. Nordtun, Ingebjørg Helena Nymo, Skarphéðinn G. Þórisson, Tiina Reilas, Ulrika Rockström, Javier Sánchez Romano, Rán Thorarinsdottir, Morten Tryland, Jonas Johansson Wensman, Ann Albihn

**Affiliations:** a National Veterinary Institute, Uppsala, Sweden; b Yakutian Research Institute of Agriculture, Yakutsk, Russia; c Natural Resources Institute Finland (Luke), Jokioinen, Finland; d Section for Pathology, The Norwegian Veterinary Institute, Tromsø, Norway; e Department of Arctic and Marine Biology, Arctic Infection Biology, UiT- The Arctic University of Norway, Tromsø, Norway; f East Iceland Nature Research Centre, Egilsstaðir, Iceland; g Farm and Animal Health, Uppsala, Sweden; h Department of Clinical Sciences, Swedish University of Agricultural Sciences, Uppsala, Sweden; i Department of Biomedical Sciences and Veterinary Public Health, Swedish University of Agricultural Sciences, Uppsala, Sweden

**Keywords:** BDV, BVDV, *Rangifer*, reindeer, pestivirus, serology, PCR

## Abstract

Reindeer herding is of great importance for the indigenous people of the Fennoscandia peninsula and northern Russia. There are also free-ranging feral populations of reindeer in Finland, Iceland, Norway and Russian Federation. The genus *Pestivirus* contains several viral species that infect ungulates and often show capacity to transmit between different host species. Sera from 520 Eurasian tundra reindeer (*Rangifer tarandus tarandus*) from Finland, Sweden, Norway, Iceland and Russian Federation were analysed and the prevalence of pestivirus-specific antibodies was determined. Seropositivity proportion was 48.5% for Sweden and 41.2% for Norway, but only 1.6% for Iceland and 2.5% for Finland. All Russian reindeer investigated were seronegative. Pan-pestivirus RT-PCR of seronegative animals (n = 156) from seropositive herds confirmed their negative status. These results indicate unexpectedly non-uniform circulation of an as yet uncharacterised pestivirus in Eurasian reindeer populations. The high seroprevalence in some regions warrants further studies of pestivirus infection dynamics, effects on reindeer health and population dynamics.

## Introduction

Pestiviruses are of great veterinary importance since they infect a wide variety of ungulates, leading to serious health problems that can cause considerable economic losses for the livestock industry worldwide [1]. Semi-domesticated reindeer (*Rangifer tarandus tarandus*) are herded by indigenous people in Eurasia, among whom they hold great historical, cultural, socio-economic and ecological importance [2]. Previous studies of pestivirus infection in reindeer, most recently reviewed by Larska [3], have reported the seroprevalence in Finland [4], Norway [5–7] and Sweden [8,9]. However, there is a need to update the current information. To date, there are no published data available on pestivirus prevalence among reindeer in Iceland and Russian Federation. The clinical relevance of pestivirus in semi-domestic reindeer herds and populations is largely unknown. Extensive husbandry practices make it difficult to observe disease and diseased animals often fall victim to predators. Due to the high impact of pestivirus infections on health and reproduction in other species [1], it is important to investigate how reindeer are exposed and affected.

Pestiviruses are single-stranded positive-sense RNA viruses belonging to the Flaviviridae family. Eleven species of pestiviruses are currently recognised by the International Committee on Taxonomy of Viruses. These are: Pestivirus A (formerly bovine viral diarrhoea virus type 1 [BVDV-1]), Pestivirus B (former BVDV-2), Pestivirus C (classical swine fever virus [CSFV]), Pestivirus D (border disease virus [BDV]) and newly discovered species currently grouped into Pestivirus E-K [10]. The latter have been proposed due to the ongoing discovery of novel divergent pestiviruses using next-generation sequencing (NGS) techniques [1,10]. Here, we use the old terminology to simplify comparison with previous studies.

Experimental infection of reindeer with BVDV induces a variety of clinical signs, e.g. diarrhoea and nasal discharge, as well as viraemia and seroconversion [11]. Only a single pestivirus isolate (V60-Krefeld, Reindeer-1) has been obtained to date, from a naturally infected reindeer at Duisburg Zoo, Germany, that presented with severe diarrhoea [12]. In the same zoo, an almost identical pestivirus (V65-Krefeld, Bison-1) was isolated from a European bison (*Bison bonasus*) [12,13]. It was initially believed that these viruses represented a new pestivirus species, but further genetic studies revealed a close relationship to BDV-2 strains isolated from German sheep [14]. The nature of the pestivirus species circulating in free-ranging reindeer remains unknown.

The objective of this study was to determine the current seroprevalence of pestivirus and to perform direct screening for the virus by RT-PCR in seronegative animals in Eurasian tundra reindeer (*Rangifer t. tarandus*) populations in different locations in Russian Federation, Finland, Sweden, Norway and Iceland. Better knowledge of pestivirus prevalence in different geographical areas will improve understanding of its epidemiology and clinical relevance in reindeer.

## Material and methods

### Sampling

In total, 520 serum samples from Eurasian tundra reindeer (*Rangifer t. tarandus*) herds in Finland, Sweden, Norway, Iceland and Russian Federation (Ust-Yanks District, northern Yakutia) were included in the study (Table 1, Figure 1A-B). The samples from Finland and Sweden were obtained from reindeer abattoirs, whereas samples from Norway were collected from live animals in corrals. From each of these countries, three geographic locations, reflecting different pasture and herding conditions, were selected, referred to here as ‘A’, ‘B’ and ‘C’. In Iceland, wild reindeer were sampled during hunting. Calves (≤1 year old) and adult animals (>1 year old) were both sampled. The samples were collected in two consecutive years at each site during the period November 2016-September 2018 in all countries except Russian Federation, where the sampling was performed only in December 2017. Blood was sampled during slaughter except for Norway and Iceland, where live animals were sampled when bleeding the animals. All animals sampled at slaughter were examined *ante mortem* by an official veterinarian and classified as healthy.

**Table 1.** Details of the 520 serum samples obtained from Eurasian tundra reindeer (*Rangifer t. tarandus*), including both calves (≤1 year) and adult animals (>1 year) from Finland, Norway, Sweden, Iceland and Russian Federation. Sampling was performed during two consecutive years. Samples from Finland, Norway and Sweden were taken from three geographically separated herds in each country.

	Sampling 1				Sampling 2			
Sampling site	Time of sampling	Total no. of samples	No. of calves	No. of adults	Time of sampling	Total no. of samples	No. of calves	No. of adults
Finland, A	2016–12	19	10	9	2017–11	21	13	8
Finland, B	2017–01	21	14	7	2017–10	20	10	10
Finland, C	2017–02	21	10	11	2017–10	20	10	10
Norway, A	2016–11	20	10	10	2017–11	19	10	9
Norway, B	2017–01	20	10	10	2018–04	20	10	10
Norway, C	2017–01	20	10	10	2018–01	20	10	10
Sweden, A	2016–11	19	9	10	2017–12	20	10	10
Sweden, B	2016–12	33^a^	14	9	2017–12	20	10	10
Sweden, C	2016–11	20	10	10	2017–11	20	10	10
Iceland	2017–08	25	1	24	2018–09	103	14	89
Russian Federation	2017–12	19	3	16	n/a	n/a	n/a	n/a
Total		237	101	126		283	107	176

^a^Ten of the sampled animals were of unknown age.

**Figure 1.**
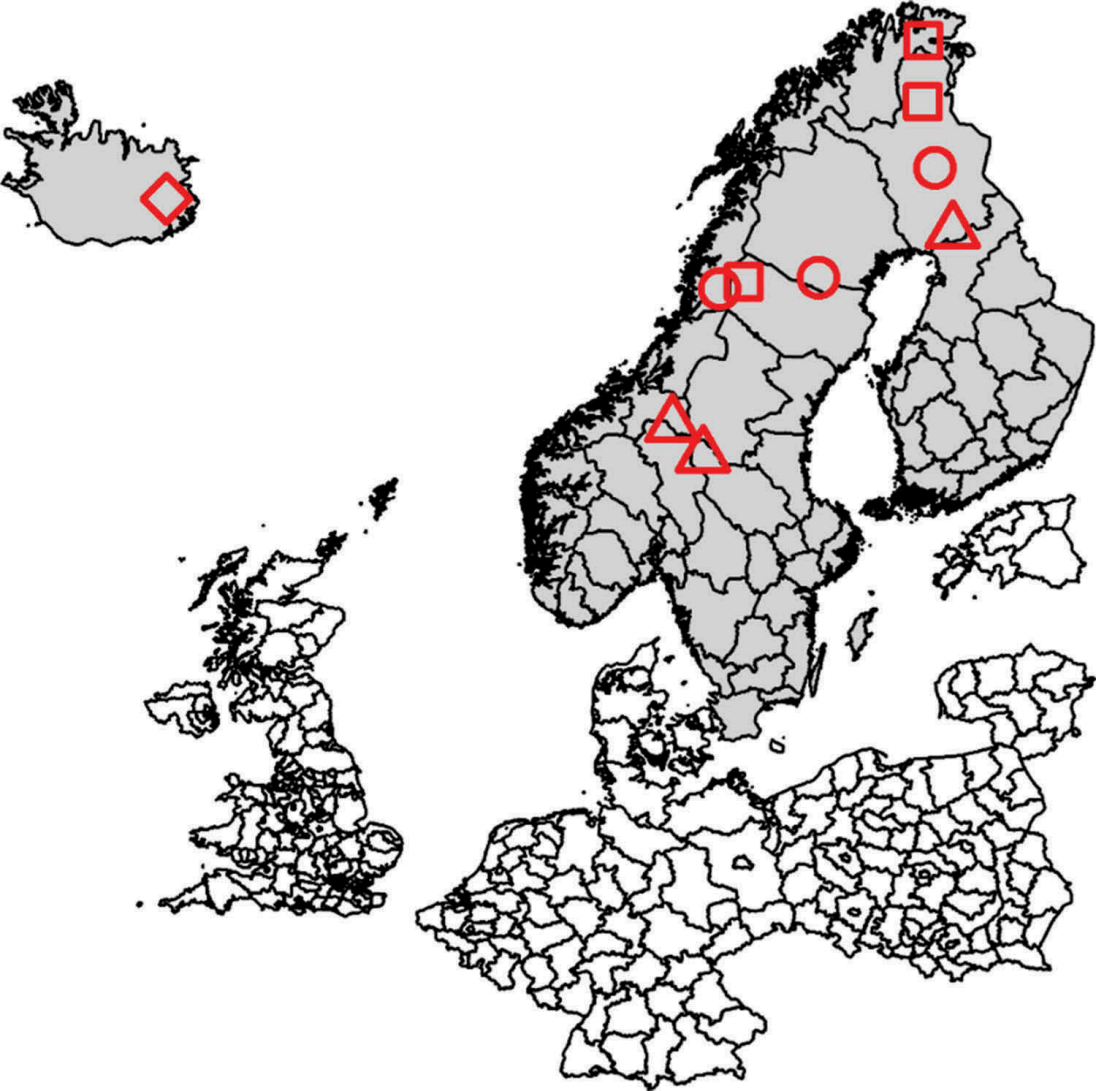
(a) Map showing the sampling sites in Finland, Sweden and Norway (reindeer abattoirs with catchment areas selected to reflect herding conditions at different locations, denoted ‘A’ (■), ‘B’ (●) and ‘C’ (▲) and the sampling sites in Iceland (♦). (b) Map showing the sampling site in Ust–Yanks District, northern Yakutia, Russian Federation (♦).

**Figure 1.**
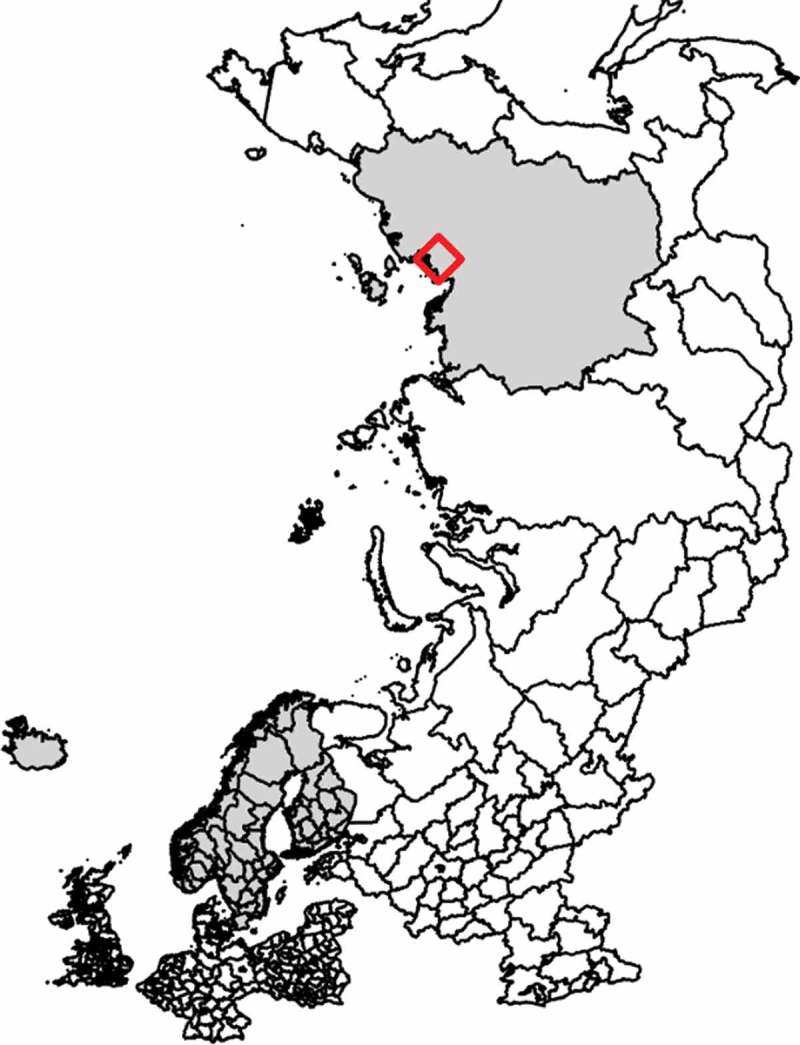
(Continued.)

### Blocking ELISA

Serum samples were analysed for pestivirus antibodies by blocking ELISA (SERELISA BVD p80 Ab Mono Blocking Kit, Synbiotics Corporation, Lyon, France) according to the manufacturer’s procedure for ovine and caprine serum samples, with the minor adaptation that samples were tested as both small ruminants (1:5) and cattle (1:10). The reason for using two dilutions when analysing serum from odd species is to get rid of possible disturbing factors and to avoid dilution of weakly positive samples to below the detection limit. The assay is designed to detect specific antibodies to the non-structural protein p80/125, which is conserved for all strains of BVDV and BDV [15]. The optical density (OD) was measured at 450 nm and competition percentage for each sample was calculated. A sample with competition percentage above 40% in either of the two dilutions (1:5 and 1:10) was considered positive. Samples with competition percentage between 20% and 40% in both dilutions were considered doubtful (undetermined). Samples with competition percentage below 20% in at least one of the dilutions (i.e. both dilutions below 20% or one below 20% and one undetermined) were considered negative.

### Virus neutralisation tests (VNT)

To confirm that results of the blocking ELISA are antibodies to a pestivirus and not unspecific reactions, 15 samples classified as positive or doubtful in the ELISA test were further analysed by virus neutralisation test (VNT) using the cytopathic BVDV-1 strain Borgen Ug-59 at a concentration of 100 TCID50. This strain is used as a kit independent control in the blocking ELISA at SVA. The two samples with highest measured OD from each sampling site with positive or doubtful animals during 2016 to 2017 were selected.

The VNT was carried out in 96-well cell culture plates (NUNC microwell 96F, NUNC 67,008, Thermo Scientific, Denmark). A commercial BVD antiserum (RAB0042, APHA, Surrey, UK) was used as the positive control and serum originating from certified BVDV-negative cattle was used as the negative control. The sera were heat-inactivated at 56°C for 30 min. Each sample was tested in duplicate for cytopathic effects (CPE) on the cells. The serum samples were serially diluted five-fold from 1:5 to 1:78,125 in Eagle’s minimum essential medium with tricin (EMEM; art. no. 991,460 National Veterinary Institute (SVA), Uppsala, Sweden). To ensure correct virus concentration, the working dilution was additionally diluted 10-, 100- and 1000-fold. Viral solution was added to the serum serial dilutions and incubated at 37°C with 5% CO_2_ for 60 min. Bovine turbinate (TB) cells (produced in-house at Dept. of Microbiology, SVA) in EMEM with 20% foetal bovine serum (Gibco, ThermoFisher Scientific, Carlsbad, CA, USA) were added to all wells, and plates were incubated at 37°C with 5% CO_2_ for 5 days. After the incubation period, the results were read under an Olympus light microscope. Samples were considered positive if there was inhibition of CPE in at least one well. Antibody titre was determined for each sample according to the Kärber method [16].

### RNA extraction and real-time RT-PCR

Pestivirus infection *in utero* can lead to life-long persistent infection in other ruminants, characterised by antibody-negative, viraemic and virus-excreting animals [17]. It is reasonable to expect the same type of intrauterine infections leading to persistent infection in reindeer as it is in other ruminants. In order to identify persistently infected (PI) animals, we selected seronegative samples from animals in seropositive reindeer herds, or herds with doubtful serological status, for detection of pestivirus RNA by real-time RT-PCR.

Nucleic acids from 156 serum samples from 2016 and 2017 (Sweden n = 37, Norway n = 36, Finland n = 60 and Iceland n = 23) were extracted using a Bullet Stool kit (Diasorin, Stillwater, MN, USA) and Magnatrix 8000+ extraction robot (Magnetic Biosolutions, Stockholm, Sweden). Magnetic beads were used for isolation and purification of the viral nucleic acids prior to analysis for pestivirus RNA by real-time RT-PCR. For the extraction, 90 µL of serum was added to separate wells on a 1.2 mL square well storage plate (Thermo AB-1127) together with 10 µL ≥800 U/mL proteinase K (Sigma-Aldrich, Saint Louis, MO, USA), and run in the extraction robot on a protocol modified for using the kit on the Magnatrix 8000 + . The modification involved an extra post-lysis wash using a 20% dilution of the lysis buffer provided in the kit, and two washes with wash buffers 1 and 2, respectively. Positive and negative controls, which were also used as controls in the real-time RT-PCR, were included in each extraction. All samples were analysed by real-time RT-PCR on the day after extraction.

An AgPath-ID One Step RT-PCR kit (Applied Biosystems, ThermoFisher Scientific) was used, with forward primer BVD190-F [18], reverse primer V326 [19] and the TaqMan probe TQ-Pesti [20], which is considered a pan-pestivirus RT-PCR assay. Primers and probe were synthesised by IDT (Integrated DNA Technologies), Loewen, Belgium. Two µL of extracted nucleic acids and 13 µL mastermix were used per well. The mastermix comprised 7.5 µL AgPath 2X RT-PCR buffer, 0.6 µL forward primer (10 µM), 0.6 µL reverse primer (10 µM), 0.2 µL probe (10 µM), 0.6 µL AgPath 25X RT-PCR enzyme mix and 3.5 µL nuclease-free H_2_O. The PCR program was as follows: reverse transcription at 45°C for 10 min, initial enzyme activation at 95°C for 10 min and 48 two-step amplification cycles at 95°C for 15 s and 60°C for 45 s. Samples with Ct-values ≤ 36 were considered positive, while samples with Ct-values >36 were reanalyzed in triplicate for confirmation.

## Results

### Serology

Specific antibodies to pestivirus were found in 49% (year 1) and 48% (year 2) of the samples from Sweden and 38% (year 1) and 44% (year 2) of the samples from Norway (Table 2). Seropositive animals were found at all sampling sites in Norway and Sweden, with the percentage of seropositive adults being higher than the percentage of seropositive calves (Table 3). At sampling site ‘C’ in Sweden, no calves were classified as seropositive, but 75% of the adult animals tested were seropositive. No specific antibodies to pestivirus were found in the samples from Finland during year 1 of sampling, while specific antibodies to pestivirus were found in 5% (n = 3) of the samples in year 2 (Table 2). One sampling site (‘B’) in Finland had zero animals classified as positive (Table 3). In Iceland, 1.6% (n = 2) of samples from adult animals (>1 year) were classified as seropositive in year 1, while no animals tested positive in year 2 (Table 2). No positives were detected among the Russian samples. The overall seroprevalence in all reindeer sampled in the study area was 22.7%. The overall seroprevalence in adults (>1 year) was 27.8% and overall seroprevalence in calves (≤1 year) was 13.0% (Table 3).

**Table 2.** Results of testing samples from Eurasian tundra reindeer (*Rangifer t. tarandus*) for anti-pestivirus antibodies in a commercial blocking ELISA: number of samples classified as positive, negative and doubtful (undetermined), and seroprevalence with 95% confidence interval (CI) per country and in sampling year 1 and 2. Doubtful results were not included in calculation of seroprevalence.

Country (year)	Positive	Negative	Doubtful	No. of samples/year	Total no. of samples/country	Seroprevalence, %, [95% CI]
Finland (1)	0	60	1	61	122	0 [0–5]^a^
Finland (2)	3	53	5	61		5 [0–10]
Norway (1)	23	36	1	60	119	38 [26–51]
Norway (2)	26	33	0	59		44 [31–57]
Sweden (1)	35	37	0	72	132	49 [37–60]
Sweden (2)	29	26	5	60		48 [36–61]
Iceland (1)	2	23	0	25	128	8 [0–19]
Iceland (2)	0	91	12	103		0 [0–3]^a^
Russian Federation (1)	0	19	0	19	19	0 [0–16]^a^
Total	118	378	24	520		23 [19–26]

^a^Confidence interval approximated by 3/(no. of samplings).

**Table 3.** Results of testing samples from Eurasian tundra reindeer (*Rangifer t. tarandus*) for anti-pestivirus antibodies in a commercial blocking ELISA, divided into age groups ‘calves’ (≤1 year) and ‘adults’ (>1 year), shown as number of animals classified as positive out of the total number of animals from each sampling site, and as a percentage.

	Calves		Adults		Total	
Sampling site	Positive/Total	%	Positive/Total	%	Positive/Total	%
Finland, A	0/23	0	1/17	5.9	1/40	2.5
Finland, B	0/24	0	0/17	0	0/41	0
Finland, C	2/20	0	0/21	0	2/41	4.9
Norway, A	1/20	5.0	8/19	42.1	9/39	23.0
Norway, B	4/20	20.0	14/20	70.0	18/40	45.0
Norway, C	5/20	25.0	17/20	85.0	22/40	55.0
Sweden, A	5/19	26.3	13/20	65.0	18/39	46.2
Sweden, B	10/24	41.7	14/19	73.7	31/53^a^	58.5
Sweden, C	0/20	0	15/20	75.0	15/40	37.5
Iceland	0/15	0	2/113	1.8	2/128	1.6
Russian Federation	0/3	0	0/16	0	0/19	0
Total	27/208	13.0	84/302	27.8	118/520	22.7

^a^Ten of the sampled animals were of unknown age, and seven of them tested positive.

### Virus neutralisation test

The VNT confirmed the positive results obtained in blocking ELISA for samples from Norway and Sweden, with neutralising antibody titres within the range 1:25–1:1400 (Table 4). A sample from Finland in 2017 with an ‘undetermined’ result in blocking ELISA tested negative in VNT. The two ELISA-positive Icelandic samples found in 2017 caused toxic CPE (tCPE; easily distinguish from viral CPE) in the serum controls. However, one of these samples had wells with intact cells and no viral replication in serum concentration 1:125 and lower. which indicates a positive result. The other sample showing tCPE in all wells could not be analysed in the VNT. For confirmation, the Icelandic ELISA-positive samples were run again in both Sweden and Norway, with the same result. In contrast to samples from other countries, the samples from Iceland were taken during hunting.

**Table 4.** Results of testing a subset of serum samples from Eurasian tundra reindeer (*Rangfer t. tarandus*) for anti-pestivirus antibodies in a virus neutralisation test (VNT) in herds with ELISA-positive or doubtful animals: presence of neutralising antibodies to pestivirus and titre for two animals (a and b) from one herd as determined in the VNT, compared with competition percentages from the blocking ELISA (22.7% positive, 4.6% doubtful and 72.7% negative).

Sample (site)	Positive/Negative, VNT	Antibody titre result, VNT	Competition %, ELISA
Finland, a (A)	Negative	<1:5	27.9
Norway, a (A)	Positive	1:625	102.3
Norway, b (A)	Positive	1:125	99.3
Norway, a (B)	Positive	1:25	101.4
Norway, b (B)	Positive	1:125	97.5
Norway, a (C)	Positive	1:280	98.1
Norway, b (C)	Positive	1:1400	98.9
Sweden, a (A)	Positive	1:280	87.5
Sweden, b (A)	Positive	1:625	77.0
Sweden, a (B)	Positive	1:56	93.0
Sweden, b (B)	Positive	1:280	88.5
Sweden, a (A)	Positive	1:280	82.5
Sweden, b (B)	Positive	1:625	86.0
Iceland, a	Positive^a^	>1:78,125	55.9
Iceland, b	N/D^a^	N/D	50.4

^a^Both sera from Iceland caused cytopathogenic effects in the serum controls. In serum Iceland 1a, the cytopathogenic effect disappeared in dilution 1:56 and at higher dilutions the virus was inhibited until >1:78,125.

### Real-time RT-PCR

All antibody negative samples from the sampling year 1 (n = 156) were analysed by real-time RT-PCR and tested negative for pestivirus nucleic acids. Positive and negative control samples were confirmed to be positive and negative, respectively.

## Discussion

Seroprevalence in Finnish reindeer herds was zero in the first year of sampling and 5% in the second year. In contrast, seroprevalence of 58% (n = 300) has been reported in a previous study in Finland [4]. However, these samples were obtained from other reindeer herds in other regions (Kemijärvi and Savukoski) in Finnish Lapland than the present study. There were also more than 30 years between the previous and present study, in which time the pestivirus infection may have cleared from the reindeer population in Finland, as described for cattle herds [21]. It is also important to note that Finland has eradicated BVDV from its cattle population since the previous study, as have Sweden and Norway [3]. However, the BVD-free status of cattle in Sweden and Norway has not affected pestivirus seroprevalence in reindeer in those countries, where the overall seroprevalence detected in the present study was similar or higher than previously reported [5,7,9,17,22]. Overall, the significance of BVDV eradication in cattle for pestivirus infections in reindeer is unclear, since the nature of these pestiviruses is unknown.

The overall prevalence (sampling years 1 and 2, calves and adults) in Norway and Sweden was 41.2% and 48.5%, respectively. Differences were found between samples classified as positive for calves (<1 year) and adult animals (≥1 year), with a higher percentage of positive adult animals at all sampling sites in Norway and Sweden (Table 3). When interpreting these results, potential presence of colostral maternal antibodies should be considered. According to Løken [23], maternal antibodies to BDV may remain in lambs for up to six months after birth. If pestivirus-specific maternal antibodies persist in reindeer for a similar period, this could have affected our results. No birth data are available but, given that most reindeer calves are born in May, some of the young animals may have been about six months of age at the time of sampling. Therefore, the seroprevalence (caused by viral infection) might have been somewhat overestimated in calves in the present study.

We found no evidence of persistently infected (PI) animals. To identify PI reindeer, sampling should be targeted at young animals showing clinical signs, e.g. retarded growth rate, assuming that PI reindeer show similar symptoms to PI cattle. There are reports of PI white-tailed deer [24], but there is still no evidence of the existence of PI reindeer. If PI reindeer exist, they would possibly be eliminated by predators if not fit enough to follow the herd. In future research on pestivirus in reindeer, it would be interesting to investigate animals with relevant clinical signs or animals found dead, and specifically search for growth-retarded PI calves. This should be done in populations with high seroprevalence among calves, where PI reindeer are more likely to be found. This would be a challenging task because of the free-ranging nature of reindeer herding, but it could yield important information regarding infection pressure in reindeer populations and how to reduce it. It could also provide information regarding whether pestivirus infection in reindeer is of clinical relevance.

No pestivirus RNA was found in real-time RT-PCR analysis of samples with negative or undetermined results from blocking ELISA. This is in accordance with previous results [9].

This study is the first to detect antibodies to pestivirus in Icelandic reindeer, and thus indicate that pestiviruses may have recently spread to the isolated Icelandic reindeer population. The two seropositive reindeer in Iceland were adults and no antibodies were detected in the 15 calves sampled. More comprehensive studies would be needed to obtain information on ongoing circulation of pestivirus in the Icelandic reindeer population.

In subarctic regions, it is likely that climate change will affect the prevalence and distribution of infectious diseases among wild, feral ranging and semi-domesticated animals such as reindeer. However, we did not find any influence of differences in locations for samples collected in Finland, Norway and Sweden. Further, the selection of sampled individuals may underestimate the pestivirus prevalence in certain populations. Most of the samples were from slaughtered animals, which had been examined and considered healthy *ante mortem*. Samples from Norway were obtained from live animals, with no previous selection for slaughter, but the samples from Iceland were taken from hunted reindeer, also selected for being apparently healthy and well-fed (animals were chosen from a long distance in a short period before shooting). Moreover, the time between death of the animal and sampling of blood from the Icelandic reindeer was usually less than 1 hour but was sometimes several hours, which might be a factor affecting the VNT serum control cells. The blood samples taken in connection with slaughter were collected immediately after death.

For Russian Federation, we only had a small number of sampled animals and the seropositive animals might have been found if randomly selected live animals had been sampled.

Therefore, some caution is necessary when interpreting our results. A positive result is best interpreted as indicating that pestivirus is present in the specific population, while a negative result cannot rule out the possibility of pestivirus prevalence.

## Conclusions

This study confirmed circulation of pestivirus in reindeer from Finland, Sweden and Norway, and for the first time, detected pestivirus in samples taken from reindeer in Iceland. The nature of the reindeer pestivirus is still unknown, as is the clinical relevance of pestivirus infection in reindeer. There is no evidence that the BVD status of cattle in a geographical area has any influence on pestivirus prevalence in reindeer.
